# Draft Genome Sequence of *Trypanosoma equiperdum* Strain IVM-t1

**DOI:** 10.1128/MRA.01119-18

**Published:** 2019-02-28

**Authors:** Batdorj Davaasuren, Junya Yamagishi, Daiki Mizushima, Sandagdorj Narantsatsral, Davaajav Otgonsuren, Punsantsogvoo Myagmarsuren, Badgar Battsetseg, Banzragch Battur, Noboru Inoue, Keisuke Suganuma

**Affiliations:** a National Research Center for Protozoan Diseases, Obihiro University of Agriculture and Veterinary Medicine, Obihiro, Hokkaido, Japan; b Laboratory of Molecular Genetics, Institute of Veterinary Medicine, Mongolian University of Life Sciences, Ulaanbaatar, Mongolia; c Department of Collaboration and Education, Research Center for Zoonosis Control, Hokkaido University, Sapporo, Hokkaido, Japan; d Global Station for Zoonosis Control, GI-CoRE, Hokkaido University, Sapporo, Hokkaido, Japan; e Obihiro University of Agriculture and Veterinary Medicine, Obihiro, Hokkaido, Japan; f Research Center for Global Agromedicine, Obihiro University of Agriculture and Veterinary Medicine, Obihiro, Hokkaido, Japan; Vanderbilt University

## Abstract

Trypanosoma equiperdum primarily parasitizes the genital organs and causes dourine in equidae. We isolated a new T. equiperdum strain, T. equiperdum IVM-t1, from the urogenital tract of a horse definitively diagnosed as having dourine in Mongolia. Here, we report the whole-genome sequence, the predicted gene models, and their annotations.

## Abstract (precis)

Trypanosoma equiperdum primarily parasitizes the genital organs and causes dourine in equidae. We isolated a new T. equiperdum strain, T. equiperdum IVM-t1, from the urogenital tract of a horse definitively diagnosed as having dourine in Mongolia.

## ANNOUNCEMENT

Trypanosoma equiperdum belongs to the kingdom *Protista*, phylum *Sarcomastigophora*, class *Zoomastigophorea*, order *Kinetoplastida*, family *Trypanosomatidae*, genus *Trypanosoma*, subgenus *Trypanozoon* together with T. brucei and T. evansi (1). The taxonomy of *Trypanozoon* trypanosomes has been controversial because of their close evolutionary relationship and insufficient genetic markers. Despite the fact that many isolates taxonomically characterized as T. equiperdum were isolated over the past 50 years, it has been hypothesized that almost all of them likely represent misclassified T. evansi or T. brucei isolates (2). Recently, some T. equiperdum strains were newly isolated from Italy (strain ICT2011) (3), Ethiopia (strain Dodola) (4), and Venezuela (strains TeAp-N/D1 and TeGu-ND1) (5). Among the *Trypanozoon* group, whole-genome sequences of T. brucei strain TREU927, T. evansi strain STIB805, and T. equiperdum strain OVI were published in 2005 (6), 2015 (7), and 2017 (8), respectively. Here, we report the draft whole-genome sequence of the culture-adapted T. equiperdum strain IVM-t1, which was isolated from the urogenital tract of a stallion definitively diagnosed as having dourine in Mongolia (9).

The T. equiperdum strain IVM-t1 isolate was cultivated using Hirumi's modified Isocove's medium-9 (HMI-9) soft-agarose medium (HMI-9 with 0.8% low gelling agarose [type VII, Sigma-Aldrich Japan, Tokyo, Japan]) at 37°C in 5% CO_2_ (9). The total DNA of culture-adapted T. equiperdum IVM-t1 was extracted and purified using Tris-EDTA (TE)-saturated phenol (Sigma-Aldrich Japan) and phenol-chloroform isoamyl-alcohol solution (Sigma-Aldrich Japan) (10). Purified DNA was kept at –30°C until use. The genome libraries of T. equiperdum IVM-t1 were prepared using a MiSeq reagent kit v3 for MiSeq sequencing (Illumina, Inc., CA), a DNA/polymerase binding kit P6 v2, and a DNA sequencing kit 4.0 v2 for PacBio RS II sequencing (Pacific Bioscience, Inc., CA). The MiSeq sequencer produced 28,337,050 paired-end reads with an average read length of 300 bp, and the PacBio RS II sequencer produced 7,508 reads with an average read length of 8,523 bp (range, 500 to 42,071 bp). The low-quality reads were trimmed using FastQC v0.11.5 and FASTX-Toolkit v0.0.13.

Whole-genome assembly was performed with ABySS-2.0.2 (11) with parameter settings of –pe, np = 12, and k = 68 for MiSeq reads and with Hierarchical Genome Assembly Process 3 (HGAP.3) (12) at the default settings for PacBio reads. The MiSeq short reads were aligned to the contigs produced from PacBio reads using Bowtie v1.2.1.1 (13) with parameter settings of -p 12, -x, and -S. The quality of the contigs, which were derived from PacBio aligned with MiSeq, were improved using Pilon v1.21 (14) with parameter settings of -Xmx128G, –changes, –vcf, and –tracks. The obtained contigs were integrated into the draft genome of T. equiperdum by Metassembler v1.5 (15) with the parameter settings bowtie2_maxins = 600, bowtie2 minins = 0, and meta2fasta_keepUnaligned = 0. The chromosome construction, gene prediction, and gene annotation of each predicted gene of T. equiperdum IVM-t1 were performed using the published genome of T. brucei brucei (T. b. brucei) TREU927 as a reference via the Companion pipeline (https://companion.sanger.ac.uk/) (16) with default settings and without transcript evidence.

The integrated draft genome consisted of 45 contigs with an *N*_50_ value of 859,849 bp and a cumulative length of 26,988,997 bp (≈27 Mbp). The T. equiperdum IVM-t1 draft genome contains 7,718 protein-coding genes, 102 noncoding genes, and 2,473 pseudogenes. Following comparison of the predicted genes of T. equiperdum IVM-t1 with the reference gene sets of T. b. brucei TREU927 using OrthoMCL v2.0.7 (17), 6,831 protein-coding genes shared between the two species and 880 T. equiperdum IVM-t1-specific protein-coding genes were identified. Seven short genes that have less than 25 amino acids were not included in the comparison (https://doi.org/10.6084/m9.figshare.7552262.v1).

In conclusion, the whole-genome draft assembly produced in the present study provides a resource for future trypanosome genetic studies and identifies some T. equiperdum-specific genes.

### Data availability.

This whole-genome project has been deposited in DDBJ/ENA/GenBank under the accession no. QSBY00000000. The version described in this paper is the first version, QSBY01000000. Raw sequence reads have been deposited in the NCBI SRA database under the accession no. PRJNA477427.
